# A Cell-Based Electrochemical Biosensor for the Detection of Infectious Hepatitis A Virus

**DOI:** 10.3390/bios14120576

**Published:** 2024-11-27

**Authors:** Dilmeet Kaur, Malak A. Esseili, Ramaraja P. Ramasamy

**Affiliations:** 1 Nano Electrochemistry Laboratory, College of Engineering, University of Georgia, Athens, GA 30602, USA; dilmeet.kaur@uga.edu; 2 Center for Food Safety, University of Georgia, Griffin Campus, Griffin, GA 30223, USA

**Keywords:** cell-based biosensors, infectious viruses, Hepatitis A virus, infectivity assay, FRhK-4 cells, foodborne outbreaks, impedance spectroscopy, screen-printed electrodes

## Abstract

Hepatitis A virus (HAV), a major cause of acute liver infections, is transmitted through the fecal–oral route and close contact with infected individuals. Current HAV standardized methods rely on the detection of virus antigen or RNA, which do not differentiate between infectious and non-infectious HAV. The objective of this study was to develop a prototype cell-based electrochemical biosensor for detection of infectious HAV. A cell culture-adapted HAV strain (HM175/18f) and its permissive cells (FRhK-4), along with gold nanoparticle-modified screen-printed electrodes, were used to develop the biosensor. Electrochemical impedance spectroscopy was used to quantify the electrical impedance signal. Nyquist plots showed successful fabrication of the cell-based biosensor. The optimum period of HAV incubation with the biosensor was 6 h. A significant linear relationship (R^2^ = 0.98) was found between the signal and a 6-log range of HAV titers, with a limit of detection of ~5 TCID_50_/mL (tissue culture infectious dose). The biosensor did not detect non-target viruses such as feline calicivirus and human coronavirus 229E. The biosensor was stable for 3 to 7 days at an abusive temperature (37 °C), retaining ~90 to 60% of the original signal, respectively. In conclusion, this prototype cell-based biosensor is capable of rapidly detecting low levels of infectious HAV.

## 1. Introduction

Globally, hepatitis A virus (HAV) is the most common cause of acute liver disease [1]. The virus is primarily spread through the fecal-oral route, contaminated food, fomite and water, or by direct person-to-person contact. Fever, fatigue, loss of appetite, nausea, abdominal discomfort, dark urine, and jaundice are some of the symptoms of an HAV infection [2]. Hepatitis A virus is a small (27–32 nm), non-enveloped virus belonging to the *Picornaviridae* family. It has a single-stranded RNA genome of approximately 7.5 kb in length. The virus is classified into six genotypes, with genotype I, II, and III infecting humans [3,4]. All six HAV genogroups belong to one serotype, exhibiting little antigenic diversity [1].

Over the past decade, the United States has experienced significant HAV outbreaks, primarily through person-to-person transmission [5,6]. Since 2017, these outbreaks have predominantly affected the homeless populations and people who use drugs, leading to widespread and costly public health interventions [7,8]. In addition to person-to-person transmission, HAV causes foodborne outbreaks of significant impact on the food industry [9]. For example, in 2019, an outbreak linked to imported fresh blackberries resulted in multiple infections across several states, underscoring the risks associated with contaminated produce [10]. Similarly, a multistate outbreak in 2023, linked to frozen organic strawberries, highlighted the issues arising from contaminated fruit sourced from regions with poor sanitary practices [11]. Foodborne outbreaks of HAV have substantial impact on the global public health resulting in hospitalization rate of 0–86% and mortality rate 0–7%, depending on the country [12]. Also, the economic burden of HAV outbreaks is substantial. For example, in the United States, HAV hospitalization resulted in a USD 300 million loss between 2016 and 2020 [8]. Preventing foodborne HAV requires comprehensive food safety measures, including proper hygiene and sanitation during food handling and preparation, using clean water, and cooking food to safe temperatures. In addition to these measures, testing food, water or food-contact surfaces for the presence of HAV can help prevent, trace and respond rapidly to HAV-associated foodborne outbreaks.

Traditional detection methods for HAV, such as serologic tests and enzyme immunoassays (EIA), have been the cornerstone of diagnostic clinical practices. Serologic tests detect antibodies (IgM and IgG) against HAV in the blood of infected patients. However, these tests cannot differentiate between acute and chronic infections and may not be able to detect the virus in the early stages of infection [13,14]. Point-of-care HAV tests are another innovation, providing rapid diagnostic results at the point of care without the need for complex equipment. These tests are quick and easy to use, making them ideal for immediate testing scenarios, but they generally offer lower sensitivity and specificity compared to laboratory-based tests, which can be a drawback in ensuring accurate diagnosis [14]. Molecular detection methods such as reverse transcription-quantitative polymerase chain reaction (RT-qPCR) offer high sensitivity and specificity and are capable of detecting HAV during the early stages of infection, even before antibody production by the immune system [15,16,17]. The FDA (Food and Drug Administration) standardized method for detecting HAV in food is also based on RT-qPCR [18]. The RT-qPCR assays are expensive, require sophisticated laboratory equipment, and necessitate technical expertise [17]. Furthermore, they cannot differentiate between infectious and non-infectious viruses. Traditional cell culture infectivity assays are required to confirm virus infectivity. However, wild-type HAV recovered from human samples requires weeks to grow in cell culture [3]. In addition, cell culture-adapted HAV strains require 10–14 days of incubation to confirm infectivity [19]. For foodborne HAV, the inability to efficiently confirm HAV infectivity in cell cultures further complicates decision-making when the viral RNA is detected in food or water.

Biosensors could revolutionize the detection of viruses by offering rapid, accurate, and sensitive diagnostic capabilities directly at the point of care. Biosensors can be designed to detect specific viral components with high precision, reducing the time needed for diagnosis and allowing for quicker public health responses [20,21,22]. Electrochemical biosensors have been extensively studied and recognized for their efficacy in detecting foodborne pathogens at very low concentrations [23,24,25,26,27]. In the case of viruses, they can directly measure viral particles by detecting changes in electrical impedance resulting from the interaction between the virus and specific biological receptors. Previous studies on the use of electrochemical biosensors for the detection of HAV are limited to either the detection of HAV nucleic acid [28] or HAV antibody [29], both of which cannot confirm virus infectivity. Cell-based biosensors generate a quantitative response to a specific stimulus and are gaining attention for environmental and biomedical analyses [30]. These biosensors enable the analysis of basic cellular functions such as receptor–ligand interactions [30]; thus, they are ideal for use to detect cytopathic effects (CPEs) induced by infectious viruses. In addition, cell-based biosensors allow for more realistic interactions to be monitored because viral receptors on cells are in their native environment leading to optimal activity and specificity against viruses [31]. However, few previous studies explored the use of mammalian cell-based biosensor for determining virus infectivity [32,33,34]. For example, an earlier study used hamster kidney fibroblast cells (BHK-21) that were immobilized on a screen-printed carbon electrode, modified with poly-L-lysine to study dengue virus CPE over time [32]. Using electrochemical impedance spectroscopy (EIS), the study reported a faster detection of CPE for dengue virus (30 h) in comparison to traditional cell culture infectivity assays (72 h). Moreover, the cell-based biosensor allowed for the rapid detection of the presence of infectious viral particles from dengue-infected patient samples [32]. This rapid detection capability is crucial for effective public health interventions, especially during outbreaks.

The objective of this study was to develop a prototype electrochemical cell-based biosensor for the rapid detection of infectious HAV. The biosensor employs FRhK-4 epithelial cells as biological receptors, integrated with a bio-nanocomposite-modified electrode. The electrode is enhanced with gold nanoparticles, which increase the surface area and improve cell adhesion and signal strength. The efficacy of this biosensor was tested using EIS, demonstrating high sensitivity and specificity in detecting infectious HAV with a LOD (Limit of Detection) of ~0.7 log equivalent to 5 TCID_50_/mL. In summary, this study introduces a novel approach to detect infectious HAV which is an improvement to traditional cell culture infectivity assays. The cell-based biosensor developed here is a proof-of-concept toward developing rapid methods for the detection of infectious foodborne viruses for food safety monitoring.

## 2. Materials and Methods

### 2.1. Chemicals and Apparatus

The chemicals used in this study include Dulbecco’s modified Eagle’s medium (DMEM) (Thermo Fisher Scientific, Waltham, MA, USA); Avantor^®^ Seradigm select-grade USDA (United States Department of Agriculture); approved origin fetal bovine serum (FBS) (VWR, Radnor, PA, USA); penicillin: streptomycin 100X for tissue culture (VWR Life Science, Radnor, PA, USA); trypan blue 0.4% in an aqueous solution ready-to-use (VWR Life Science, Radnor, PA, USA); trypsin (0.25%) EDTA-1X (ethylenediaminetetraacetic acid) (VWR Life Science, Radnor, PA, USA), 20 nm gold nanoparticles supplied in 0.1 mM PBS (phosphate-buffered saline), 95% OD1 (optical density), 524 nm absorption (Thermo Fisher Scientific, Waltham, MA, USA); potassium ferrocyanide (K_4_[Fe(CN)_6_]) (Thermo Fisher Scientific, Waltham, MA, USA); potassium ferricyanide (K_3_[Fe(CN)_6_]) (VWR Life Science, Radnor, PA, USA); sodium phosphate dibasic anhydrous (Na_2_HPO_4_) (EMD Millipore, Burlington, MA, USA); sodium chloride (NaCl) (Thermo Fisher Scientific, Waltham, MA, USA); potassium chloride (KCl) (VWR Life Science, Radnor, PA, USA); and potassium phosphate monobasic anhydrous (KH_2_PO_4_) (RPICorp, Mt Prospect, IL, USA).

Phosphate-buffered saline (PBS, 10X) (100 mL) was prepared using the following chemicals and mixing them: 1.4 g of Na_2_HPO_4_, 8 g of NaCl, 0.2 g of KCl, 0.245 g of KH_2_PO_4_. Then, PBS (1X) (pH 7.4) was prepared by diluting the 10X PBS buffer with autoclaved deionized water (DIW). Milli-Q water (resistivity = 18 MΩ.cm) was used to prepare all the media and chemicals. Prepared buffers and media were sterilized by autoclaving at 121 °C and 15 psi for 30 min before the experiments. A redox solution of 5 mM [Fe (CN)_6_]^3−/4−^ (1:1) was prepared in 0.1M KCl. Carbon screen-printed electrodes TE100 (SPE) were acquired from CHI Instruments, Inc. (Austin, TX, USA). For the TE100 electrode system used in this study, a three-electrode configuration was employed, mounted on a polypropylene (PP) base plate measuring 12.6 mm by 50 mm. The working electrode (WE) was fabricated from carbon with a diameter of 3 mm, providing a surface area of 7.1 mm^2^. The reference electrode (RE) consisted of an Ag pseudo-reference, with an area of 0.1 mm^2^, while the counter electrode (CE) was made of carbon with an area of 10.6 mm^2^. Potentiostat interface 1010E (Gamry Instruments, Warminster, PA, USA) was used for sensor measurements.

### 2.2. Cell Culture

The fetal rhesus monkey kidney (FRhK-4) epithelial-like cell line (ATCC, Gaithersburg, MD, USA), stored at −196 °C in liquid nitrogen, was carefully thawed and cultured into a T-25 flask containing 10 mL of pre-warmed (37 °C) growth media (DMEM supplemented with 10% fetal bovine serum (FBS), 100 U/mL penicillin, and 100 µg/mL streptomycin). The cells were then incubated inside a 37 °C incubator with a humidified atmosphere containing 5% CO_2_. Upon reaching 80–90% confluency, the cells were passaged to maintain optimal growth conditions. For passaging, the medium was aspirated, and the cell monolayer was washed with PBS. Subsequently, 0.25% trypsin–EDTA solution was added to detach the cells. Once the cells have detached, trypsin was neutralized with an equal volume of growth media. The cell suspension was centrifuged at 1200 rpm for 5 min, and the supernatant was discarded. The cell pellet was resuspended in fresh DMEM, and an appropriate aliquot was transferred to a new culture flask containing growth medium. The FRhK-4 cells were cultured until they reached the third passage. For experiments, cells at the third passage were used to ensure optimal growth conditions and cell consistency. Subculturing continued only until the fourth passage, beyond which cells were not used to maintain experimental accuracy and reproducibility. The cell viability was determined using the trypan blue exclusion assay, and the cells were counted using a hemocytometer.

### 2.3. HAV Generation

The HAV cell culture-adapted strain HM175/18f (ATCC) was propagated in FRhK-4 cells. The generated HAV was centrifuged at 3000 rpm for 30 min at 4 °C. Then, the supernatant containing the viruses was ultrafiltered using Amicon™ Ultra-15 Centrifugal Filter Units (100 KDa) (Millipore Sigma, Burlington, MA, USA) to semi-purify the virus from components of the cell culture lysates, as previously done [35]. The ultra-filtered HAV titer was quantified using the 50% tissue culture infectious dose (TCID_50_), as described previously [36]. The CPE was read between day 10 and 14, and the virus titer was determined to be 6.32 × 10^7^ TCID_50_/mL. Following ultrafiltration, ten-fold serial virus dilutions were prepared in DMEM without FBS to avoid interference from FBS with the biosensing platform. The prepared dilutions were then aliquoted into sterile microcentrifuge tubes and stored at −80 °C to preserve viral infectivity. All procedures involving the handling and preparation of HAV were conducted in a biosafety level 2 (BSL-2) laboratory while adhering to stringent biosafety protocols.

### 2.4. Electrode Preparation

The SPE was rinsed with deionized water (DIW) and dried at room temperature for 60 min before taking the EIS measurements on the bare electrode. The SPE electrodes were washed again using 1X PBS (pH 7.4). Gold nanoparticles (AuNP), supplied in 0.1 mM PBS, OD1, were sonicated for 30 min, and then 8 µL of AuNP was drop-cast to cover the surface of the working electrode. After air-drying, the SPEs were washed with 1X PBS buffer (pH 7.4), and the EIS measurements were taken again. Following the wash with 1X PBS buffer (pH 7.4), 10 μL of the cell suspension in DMEM at a concentration of 6 × 10^4^ cells/mL was drop-cast on the working electrode. The cell adhesion period was conducted without FBS, as the presence of FBS during the adherence and measurement phases significantly increased impedance, interfering with the accurate detection of HAV. Initially, 10 µL of cell culture media was applied to the working electrode to ensure proper contact, and additional media (0.5mL) was subsequently added to cover the surface and prevent evaporation during the period of cell adsorption. The AuNP-FRhK-4 SPEs were incubated for 8 h at 37 °C inside a 5% CO_2_ incubator. The choice of an 8 h adhesion period for FRhK-4 cells on the electrode surface was guided by a previous study that demonstrated optimal cell adhesion typically occurs within 2 to 10 h, especially at a seeding density of 10,000 cells per well [19]. This time frame ensures robust attachment before viral detection experiments. Additionally, our observations conducted under the microscope during routine subculturing of the FRhK-4 cell line, confirmed that cell adherence to surfaces was consistent and stable at around 8 h. These experimental observations aligned with the expected adhesion behavior of epithelial-like cell lines in culture, further validating the choice of the 8 h time point. After cell adsorption, the electrodes were washed with 1X PBS buffer (pH 7.4) to remove any non-adhered or dead cells before taking another EIS measurement as described below.

### 2.5. Optimization of HAV Incubation Time

**Figure 1:**
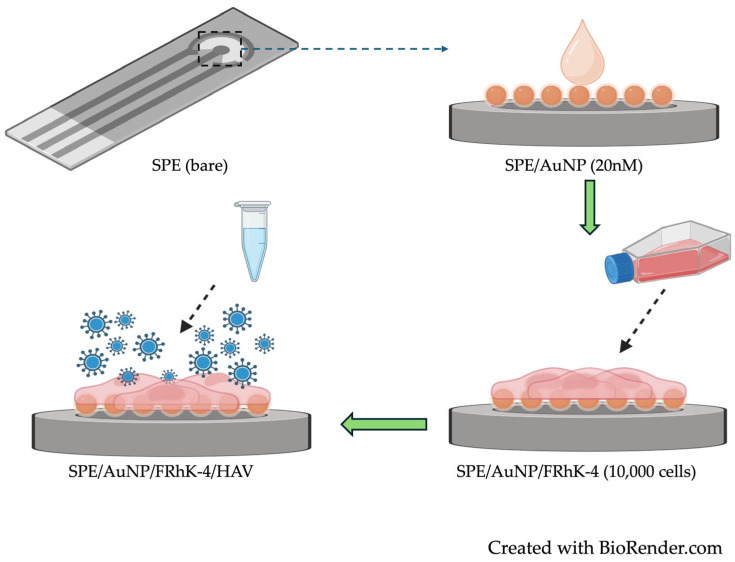
Electrode preparation steps, including the drop-cast deposition of the gold nanoparticles (AuNP) on the bare screen-printed electrodes (SPEs), followed by the immobilization of FRhk-4 cells and subsequent testing with hepatitis A virus (HAV).

The fabricated biosensor (Figure 1) was evaluated for the optimal incubation time with HAV to detect the virus binding to FRhK-4 cells. The biosensor was incubated with 10 µL of HAV at 6.32 × 10^2^ TCID_50_/mL for 0 (immediately after cell adhesion), 0.5, 1, 2, 4, 6, and 12 h inside a 37 °C incubator (5% CO_2_). After each incubation period, the virus was removed by rinsing with 1X PBS buffer (pH 7.4). Then, the EIS measurements were performed to detect the change in signal (%) relative to the initial measurement. The % signal change was calculated based on the charge transfer resistance (R_CT_) values, reflecting the interaction between HAV and the cells on the electrode surface.

### 2.6. Electrochemical Measurements

**Figure 2:**
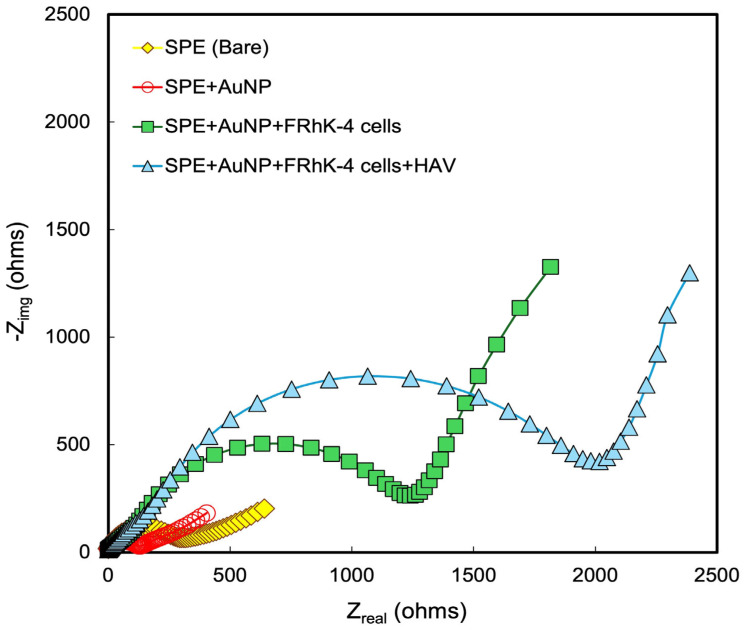
Nyquist plot of the electrode after each step of the electrode preparation process and the response of the biosensor to incubation with HAV for 6 h.

The EIS measurements were performed using the potentiostat Interface 1010E. The electrochemical system was a standard three-electrode cell using an SPE consisting of working, reference, and counter electrodes. 5 mM ${\left[Fe{\left(CN\right)}_{6}\right]}^{4-}/{\left[Fe{\left(CN\right)}_{6}\right]}^{3-}$ was used as a redox couple for the EIS measurements, and a frequency range of 1 Hz to 10 kHz with an AC amplitude of 5 mV superimposed on the open circuit potential (OCP). The resulting impedance spectra were recorded as Nyquist plots, which represent the imaginary component of the impedance (-Z_img_) as a function of the real component (Z_real_). The impedance data were modeled using an equivalent electrical circuit to interpret the Nyquist plots (Figure 2). The circuit consisted of solution resistance (R_s_) which is the resistance of the electrolyte solution; double-layer capacitance (C_dl_), capacitive behavior of the electric double layer formed at the electrode/electrolyte interface; charge transfer resistance (R_CT_), that is the resistance of electron transfer during the redox reactions at the electrode surface; and Warburg impedance (Z_w_), which is the impedance caused by the diffusion of redox species in the solution. The charge transfer resistance (R_CT_) was determined from the diameter of the semicircular arc observed in the Nyquist plot and was analyzed using the ZView v. 4 software to fit the experimental data to the equivalent circuit model, thereby extracting the (R_CT_) value.

The (R_CT_) parameter provides critical insight into the interfacial electron transfer kinetics, with larger values indicating higher resistance to charge transfer, potentially caused by surface modifications or interactions with analytes [37,38]. All measurements were performed at room temperature (298 K) under identical experimental conditions to ensure consistency.

The resulting measurement is presented as ΔR_CT_ (ohm) or ΔR_CT_ (%) where

(1) $$\Delta R_{CT}\left(\%\right)=\frac{R_{CT,measured}-R_{CT,baseline}}{R_{CT,baseline}}\times 100\%$$

(2) $$\Delta R_{CT}(ohm)=(R_{CT,measured}-R_{CT,baseline})$$

At the end of the HAV incubation period with the biosensor, the EIS measurements were carried out at room temperature (25 °C).

### 2.7. Biosensor Limit of Detection (LOD) and Specificity Testing

The LOD of the biosensor is defined as the lowest concentration of the signal detected that is different from background noise, while the limit of quantification (LOQ) indicates the lowest concentration of the signal that can be quantified with precision and accuracy. While the LOD indicates when the signal can first be distinguished from the background noise, the LOQ ensures that the signal is sufficiently reliable for accurate quantification. To determine the biosensor’s LOD, the biosensor was incubated with different concentrations of HAV, ranging from 6.32 × 10^6^ to 6.32 × 10^0^ TCID_50_/mL. The HAV volume added to the biosensor was 10 μL and the virus was allowed to incubate with the biosensor for the pre-determined optimized incubation period. The virus was removed by rinsing with 1X PBS buffer (pH 7.4) before taking another EIS measurement. Based on the relationship between the virus concentrations and the ΔR_CT_ values (the difference in charge transfer resistance), a calibration curve was obtained. The LOD and LOQ were determined based on the equations below:

$$LOD=3.3\times \left(\frac{sd}{S}\right)$$

$$LOQ=10\times \left(\frac{sd}{S}\right)$$

sd = standard deviation of the Y-intercept and S = slope of the calibration curve.

The sensitivity of the biosensor was calculated as the slope of the calibration curve divided by the area of the working electrode (0.071 cm^2^). The sensitivity of the biosensor was calculated using the following formula:

$$Sensitivity=\frac{S}{0.071}$$

The specificity of the developed biosensor was tested against feline calicivirus (FCV) and human coronavirus (HCoV 229E). Both FCV and HCoV 229E were obtained from ATCC and were generated as described previously [35,39]. Both viruses and HAV were used at a concentration of 10^2^ TCID_50_/mL and incubated with the biosensor for the same HAV pre-determined optimized period. Furthermore, FCV and HCoV 229E were also tested on a 2-day-old confluent FRhK-4 in a 96-well plate (10,000 cells/well). The cells were infected with the FCV and HCoV 299E at 10^2^ TCID_50_/mL for 7 days under similar conditions to the HAV infection of FRhK-4 cells. After the 7-day incubation period, the cells were observed for morphological changes using a bright-field inverted microscope. The control negatives include FRhK-4 cells in cell culture media but without any viruses added. Virus-induced CPEs, such as cell rounding, detachment, or lysis, were documented on day 0 and 7 using Echo Rebel (Echo—A Bico company, San Diego, CA, USA) inverted microscope-mounted camera.

### 2.8. Temperature Treatment of HAV for Testing with the Fabricated Biosensor

The HAV stock was diluted in DMEM to 6.32 × 10^4^ TCID_50_/mL. This diluted virus was divided into 1 mL aliquots which were subjected to three distinct temperature treatments: (1) incubation at 4 °C for 60 min (HAV infectious control), (2) incubation at 60 °C for 60 min (partially infectious HAV), and (3) incubation at 100 °C for 10 min (completely non-infectious virus). The biosensor was fabricated as mentioned in Section 2.4, and after the immobilization of FRhK-4 cells, triplicate biosensors were incubated with each of the HAV temperature treatments. The EIS measurements were taken and the ΔR_CT_ was calculated for each biosensor (Section 2.6).

### 2.9. Biosensor Stability

The stability of the developed cell-based biosensor was evaluated over 14 days to determine the sensor’s performance in detecting infectious HAV. Two approaches were used. First, the FRhK-4 cells were allowed to adhere to the AuNP-modified electrodes for approximately 8 h (defined as day 0) and then the cell-based biosensors were stored at 37 °C. Following the initial cell adhesion and virus exposure at day 0, subsequent virus tests were conducted on day 1, 3, 7, and 14. Second, the AuNP-modified electrodes (without the cells) were stored at room temperature for 1, 3, 7, and 14 days,; then, on each time point, the FRhK-4 cells were added and allowed to adsorb for 8 h, after which the HAV analyte was introduced. Throughout the 14-day period, the working electrode was immersed in 2 mL cell culture media in a closed petri dish to prevent evaporation of the 10 μL media during the cell incubation period. On each testing day, the biosensor was exposed to HAV titer of 10^2^ TCID₅₀/mL in 10 µL DMEM to measure its response and assess its stability. This titer was chosen because it provides a detectable signal within the biosensor’s sensitivity range without risking saturation or weak signals. The change in charge transfer resistance (ΔR_CT_) was recorded during each measurement, with ΔR_CT_ values expressed as a percentage change relative to the initial measurement taken on day 0 (immediately after the 8-h cell adhesion period).

### 2.10. Statistical Analyses

All experiments were independently repeated at least three times. Each experiment included 3 technical replicates. The statistical significance of differences between groups was assessed using a one-way ANOVA followed by Tukey’s post hoc test for multiple comparisons. Linear regression analysis was used to determine the relationship between HAV titers and signal. All data are presented as mean values of charge transfer resistance (ΔR_CT_) ± standard deviation (SD). Statistical significance was determined at *p* < 0.05. Graphpad Prism 10 and Microsoft excel were used for all statistical analyses.

## 3. Results and Discussion

### 3.1. Development of the Biosensing Platform and Optimization of Incubation Time for HAV Detection

**Figure 3:**
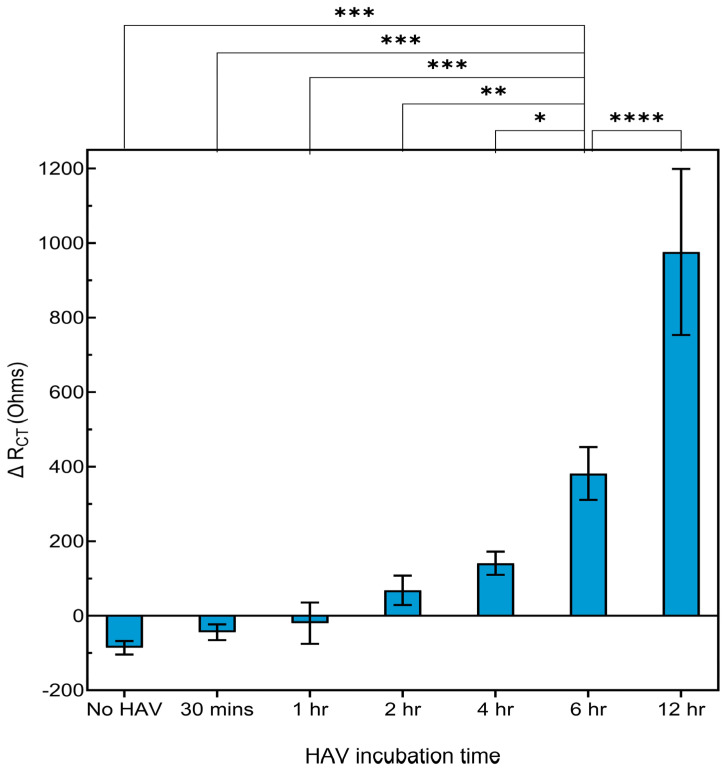
Optimization of HAV incubation time with a FRhK-4 cell-based biosensor. The % signal change detected by electrochemical impedance spectroscopy (EIS) after various HAV incubation times: 0 (immediate), 0.5, 1, 2, 4, 6, and 12 h. Data are presented as the mean ± standard deviation (SD). Statistical significance was determined using an ANOVA. Asterisks indicate significant differences at * *p* < 0.05, ** *p* < 0.01, *** *p* < 0.001, **** *p* < 0.0001.

The process of electrode modification is illustrated in Figure 1. The methodology and design of our study focus primarily on electrochemical impedance spectroscopy (EIS) as the non-destructive approach for measuring the changes in impedance resulting from the analyte (HAV) binding to cell surfaces without risking cell viability [40]. The Nyquist plot obtained from the EIS measurements (Figure 2) illustrates the imaginary impedance (-Z_img_) versus the real impedance (Z_real_) across a range of frequencies, revealing two characteristic regions: a semicircular region at higher frequencies, representing the charge transfer process, and a linear region indicative of the diffusion-controlled process. The semicircular diameter, which correlates directly with the charge transfer resistance (R_CT_), provides critical insights into electrode modifications and analyte interactions [22,41,42,43,44]. The bare electrode with AuNP showed the smallest semi-circle in the representative Nyquist plot for the biosensor, indicating the lowest charge transfer resistance of 152 Ω (Figure 2). It is known that the use of AuNPs not only enhances the electrical conductivity of the electrode [45], but also promotes strong and stable cell attachment due to the increased surface roughness and biocompatibility of the nanoparticles [46,47,48]. At a low concentration of 0.05 mg/mL, 20 nm gold nanoparticles (AuNPs) are biocompatible and non-toxic, with studies showing that concentrations up to 0.10 mg/mL maintain similar impedance patterns as the positive control and support normal cell proliferation without significant toxicity [49,50]. Thus, here, the advantages of using AuNPs for FRhK-4 cell immobilization include their excellent biocompatibility, high surface area-to-volume ratio, and enhanced electrical properties, making them ideal for developing a sensitive and reliable biosensor. In this study, the R_CT_ values showed a progressive increase across the stages of biosensor development and analyte binding, starting at the lowest for the AuNP-modified electrode, increasing significantly to 1289 Ω after immobilizing FRhK-4 cells, and finally reaching 2083 Ω upon HAV binding. This incremental rise in R_CT_ reflects the stepwise assembly and successful functionalization of the biosensor, with each modification adding resistance due to hindered electron transfer (Figure 2). The incubation time for HAV was based on pre-determined optimization shown below (Figure 3). This indicates the highest resistance, possibly because of the virus interaction with FRhK-4 cells that form additional biological layers, creating a thick interface that hinders electron transfer, leading to higher impedance.

The HAV detection time was optimized by evaluating various incubation periods of the virus with the FRhK-4 cell-based electrochemical biosensor. The results indicated minimal signal change at 30 min and 1 h, suggesting insufficient virus attachment to the cells at these early time points (Figure 3). A non-significant increase in % signal was observed at 2 and 4 h, indicating progressive interaction between HAV and the cells. The most significant signal increase was observed at 12 h; however, the 6 h time point was also significant and provided a robust and consistent signal while maintaining smaller standard deviation (Figure 3). The 6 h time point was identified as the optimal incubation time, balancing effective HAV detection with experimental efficiency, and was adopted in this study.

### 3.2. Sensitivity and LOD of the Biosensor

**Figure 4:**
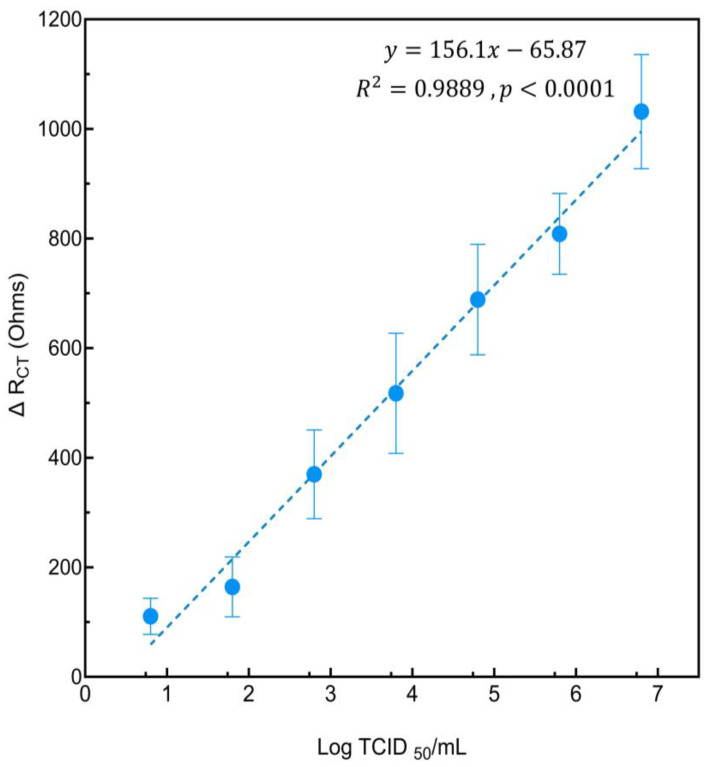
Sensitivity of the cell-based biosensor for detecting various HAV titers. The graph shows the relationship between the change in charge transfer resistance (ΔR_CT_) and HAV titers. The linear regression equation was found to be y = 156.1x − 65.872 with an R^2^ value of 0.9889 (*p* < 0.0001).

The performance of the electrochemical cell-based biosensor in detecting HAV was evaluated by measuring the change in charge transfer resistance (ΔR_CT_) across a range of HAV concentrations, from 6.34 × 10^0^ to 6.34 × 10^6^ TCID_50_/mL. A significant linear relationship was observed between ΔR_CT_ and HAV titers following the equation y = 156.1x − 65.872 and an R^2^ value of 0.9889 (Figure 4). This high R^2^ value indicates excellent linearity and reliability of this cell-based biosensor in detecting a wide range of concentrations for HAV. The sensitivity of the biosensor was found to be 2198.59 Ωcm^−2^/Log_10_ (TCID_50_/mL). This high sensitivity is attributed to the effective modification of the electrode surface with gold nanoparticles and the subsequent immobilization of FRhK-4 cells, which significantly enhanced the electrochemical response. A previous impedimetric cell-based biosensor developed for dengue virus to monitor CPE in BHK-21 cells reported changes related to viral titer and infection kinetics rather than direct sensitivity values [32]. In our study, the lowest virus titer detected for the developed biosensor was determined to be 0.8 log TCID_50_/mL (Figure 4). The LOD was calculated using the SD of Y-intercept and was found to be ~0.7 log equivalent to 5 TCID_50_/mL (based on the equation given in Section 2.7) demonstrating the sensor’s capability to detect low concentrations of infectious HAV. The LOQ was calculated to be ~2 log equivalent to 100 TCID_50_/mL (based on the equation given in Section 2.7), ensuring that the sensor can reliably quantify HAV concentrations across a broad range. This value suggests that while the sensor can detect lower viral loads at the LOD, it can only provide precise and reliable measurements at viral titers above the LOQ. In a previous study, a DNA hybridization electrochemical biosensor was developed to detect HAV, showed a LOD of 6.94 fg/µL for viral cDNA [28]. Similarly, another cell-based biosensor was developed to detect SARS-CoV-2 spike protein, resulting in an LOD of 1 fg/mL [33]. A previous study relied on HAV specific antibodies in an indirect competitive electrochemical immunosensor, including secondary antibody labeling with peroxidase, and reported a detection limit of 26 × 10⁻^5^ IU/mL [29]. The ultra-low LODs in these studies cannot be directly compared to our cell-based biosensor’s LOD, because the authors used a different approach by targeting viral genetic material or viral protein rather than the whole infectious virus. The performance of various electrochemical biosensors for HAV detection, including our current study, highlighting key parameters such as detection methods, platforms, biorecognition elements, and limits of detection (LOD) has been summarized in Table 1.

### 3.3. Specificity of the Biosensor

**Figure 5:**
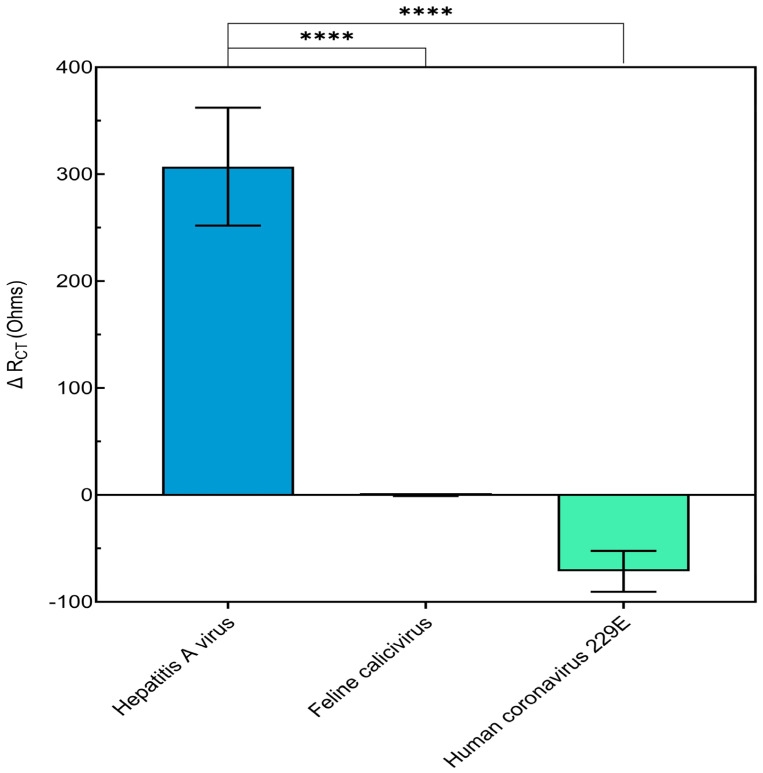
Specificity of the cell-based biosensor for detecting infectious Hepatitis A virus (HAV) compared to non-target viruses: feline calicivirus (FCV) and human coronavirus (HCoV 229E) used at 10^2^ TCID_50_/mL. Data are presented as the mean ± standard deviation (SD). Statistical significance was determined using an ANOVA. Asterisks indicate significant differences at **** *p* < 0.0001.

A significant ΔR_CT_ of approximately 300 Ohms for HAV was observed, whereas the ΔR_CT_ values for FCV and HCoV 229E were negligible, close to 0 Ohms (Figure 5). This substantial difference highlights the biosensor’s high specificity for HAV. The selection of FCV and HCoV 229E as non-specific viruses in this study was based on their relevance in different contexts: FCV is a nonenveloped virus commonly used as a surrogate for foodborne human noroviruses in food safety studies [51], while HCoV 229E, is an enveloped virus that is often used a surrogate for SARS-CoV-2, the causative agent of the COVID-19 pandemic [52,53].

**Figure 6:**
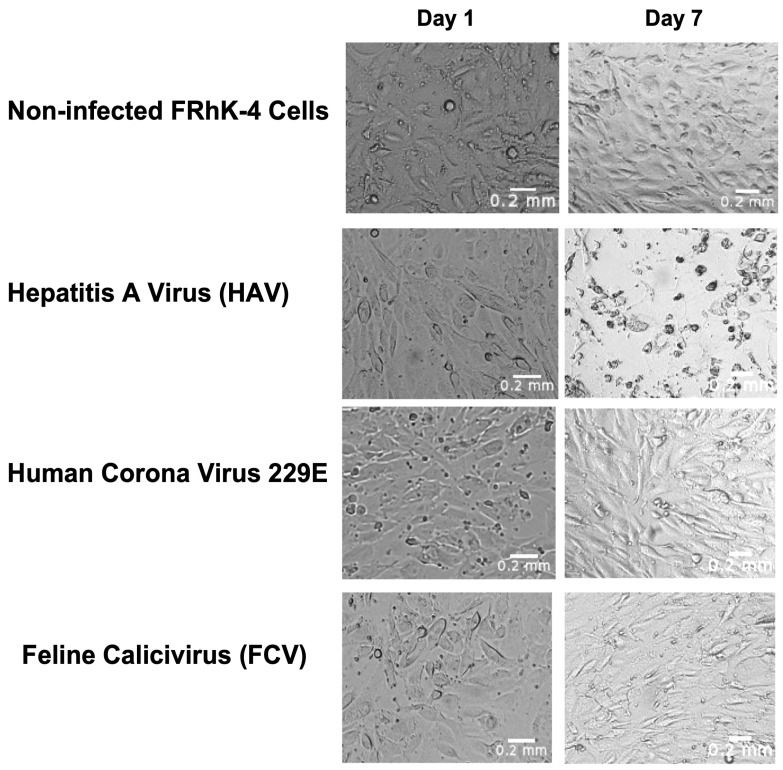
Morphological changes in FRhK-4 cells as observed under the microscope at 10× magnification. The cells were incubated with 10^2^ TCID_50_/mL infectious hepatitis A virus (HAV), feline calicivirus (FCV), and human coronavirus (HCoV 229E) over 7 days at 37 °C. The control cells were FRhK-4 cells without any added viruses.

Observations under a bright-field inverted microscope revealed significant morphological changes in the FRhK-4 cells incubated with HAV, including cell rounding, detachment, and signs of lysis, indicating virus-induced CPE (Figure 6). In contrast, the cells incubated with FCV and 229E showed no significant morphological changes, maintaining their normal morphology throughout the incubation period as compared to control non-infected cells (Figure 6). The high specificity of the biosensor to HAV lies in the use of FRhK-4 epithelial cells as a biological receptor. These cells exhibit high affinity for HAV [36], allowing the virus to bind effectively to the cells on the electrode surface. In contrast, FCV and 229E do not have the same affinity for FRhK-4 cells as also evidenced in our observations under the microscope. This results in minimal or no binding, and consequently, no significant change in ΔR_CT_. This selective binding is critical for distinguishing infectious HAV from other viruses, ensuring accurate detection and reducing false positives. While this biosensor was shown to be specific to HAV, when tested against two non-target viruses, more comprehensive testing of other viruses that can be found in the environment should be carried out in future studies. Nevertheless, this biosensor is suitable as a tool for determining the infectivity of HAV from known HAV-positive patients or HAV RT-qPCR-positive food, water, or environmental samples.

### 3.4. Differentiation Between Infectious and Non-Infectious HAV

**Figure 7:**
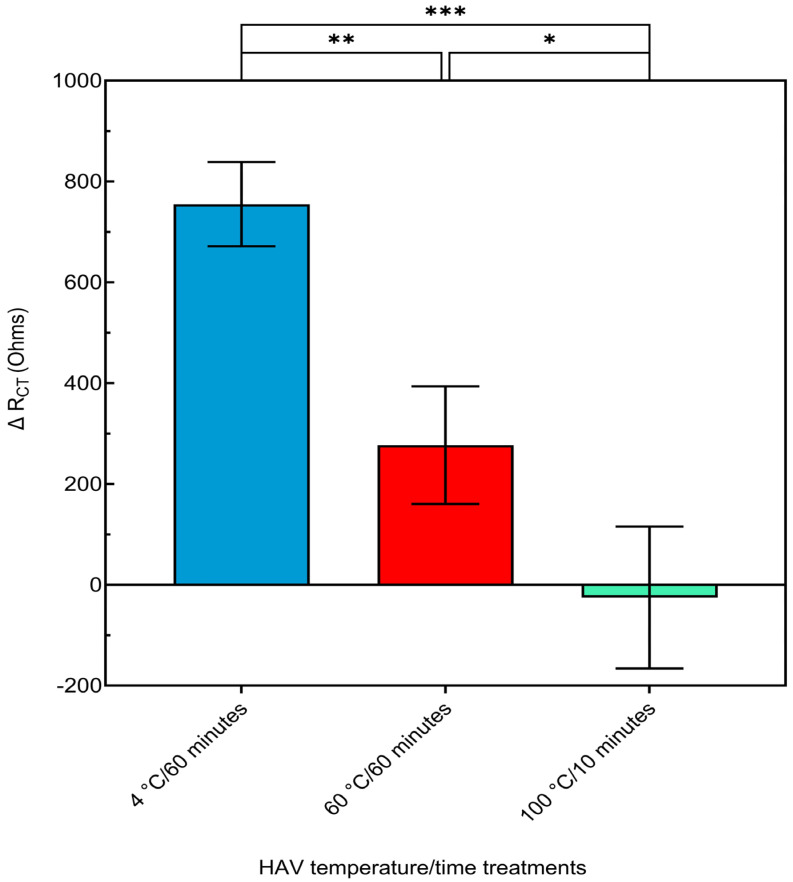
Comparison of the ΔR_CT_ values of various temperature treatments for HAV (6.32 × 10^4^ TCID_50_/mL): 4 °C for 60 min (control infectious HAV), 60 °C for 60 min (partially infectious HAV), and 100 °C for 10 min (non-infectious HAV). Data are presented as the mean ± standard deviation (SD). Statistical significance was determined using an ANOVA. Asterisks indicate significant differences at * *p* < 0.05, ** *p* < 0.01, *** *p* < 0.001.

The highest ΔR_CT_ value was observed in the control (4 °C/60 min) infectious HAV (~755 Ω) (Figure 7), which corresponds to the expected value observed earlier (Figure 4). In contrast, the 60 °C treatment group displayed a much lower ΔR_CT_ (~277 Ω) compared to the control (Figure 7). This result is expected because heat treatment at 60 °C for 60 min was shown previously to partially inactivate HAV [54], indicating that some viruses are still capable of infecting FRhK-4 on the biosensor. Finally, the 100 °C treatment group, exhibited the lowest ΔR_CT_ value (−25.4Ω), indicating negligible or no interaction with the FRhk-4 cells (Figure 7). This result is also expected because treatment at 100 °C for 10 min was shown previously to completely inactivate HAV [55], rendering the virus non-infectious and incapable of binding FRhK-4 cells to induce any impedance change. The significant differences among the groups confirm that the cell-based biosensor can accurately distinguish between infectious, partially infectious, and non-infectious HAV.

### 3.5. Biosensor Stability Testing

**Figure 8:**
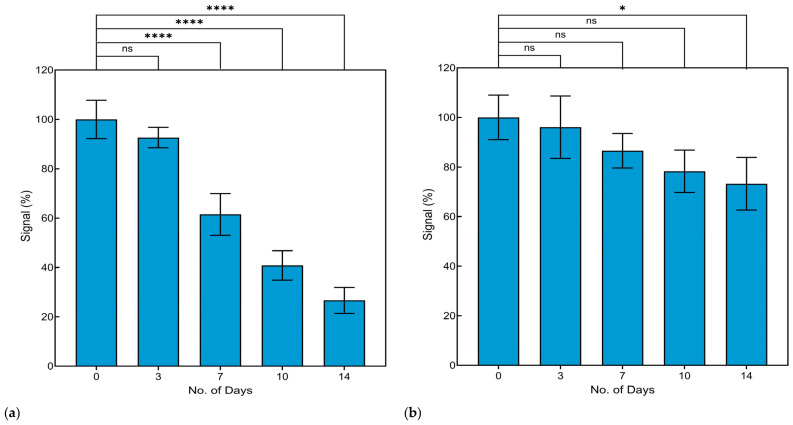
Stability of the cell-based biosensor for detecting infectious HAV. (**a**) The cell-based biosensor was stored in DMEM media over a 14 day-period inside a 37 °C incubator, then used for HAV detection on days 0, 3, 7, 10, and 14. (**b**) The AuNP-modified electrodes were stored at room temperature over a 14 day-period, but was modified with FRhK-4 cells on days 0, 3, 7, 10, and 14, followed by the addition of HAV. Data are presented as the mean ± standard deviation (SD). Statistical significance was determined using an ANOVA. Asterisks indicate significant differences at * *p* < 0.05, **** *p* < 0.0001, Non-significant differences (*p* > 0.05) compared to day 0 are indicated by the letters ns.

As depicted in Figure 8a, there is a clear decrease in the percentage of signal over the 14-day period of storage of the cell-based biosensor inside the 37 °C incubator. The signal percentage, i.e., the ΔR_CT_ values, indicate the difference between the R_CT_ of HAV-infected cells and the FRhK-4 cells. Initially, the biosensor maintained a high signal, retaining close to 100% of its ΔR_CT_ on the first day. On days 3 and 7, the signal retained was ~90 and 60%, respectively (Figure 8a). However, by day 10, the signal significantly decreased to around 20%. This reduction in signal may be attributed to the decreased viability of the FRhK-4 cells immobilized on the electrode surface in the absence of FBS. The cells’ ability to bind HAV diminishes as they lose viability, resulting in a reduced electrochemical response. Living cells are essential for maintaining the cell-based biosensor’s functionality, as they provide the biological recognition element required for specific virus binding. Over time, the cells undergo apoptosis or necrosis, which impairs their binding capabilities and, consequently, the sensor’s performance. Maintaining cell viability is a critical factor for the long-term stability of cell-based biosensors. Overall, our results showed that this cell-based biosensor, when stored under abusive temperature (37 °C), should be used within 3 days.

To explore whether storage temperature affected the AuNP-modified SPE electrode itself, another time stability experiment was conducted at room temperature on AuNP-modified SPE without initially adding the cells. The cells were added on the different storage days and then allowed to bind to HAV for 6 h. The biosensor maintained over 90% of the initial signal on day 3 (Figure 8b). On days 7 and 10, a slight decline in signal was observed, while on day 14, the retention remained above 75% (Figure 8b). The ΔR_CT_ values suggest that the AuNP-modified electrodes (without immobilized cells) can maintain their functional performance for up to 14 days at room temperature. The decrease in ΔR_CT_ over time may reflect minor changes in the surface characteristics of the modified electrodes; despite these changes, the electrodes consistently provided a strong response, suggesting robustness for real-time viral detection applications. Therefore, to improve the stability, future research should focus on further optimizing the storage temperature and incorporating cell-preserving agents or essential growth factors such as FBS. Additionally, exploring alternative cell lines or engineering more robust cells might further enhance the stability of this cell-based biosensor.

**Table 1:** Comparison of electrochemical biosensors for HAV detection.

Method	Platform	Biorecognition Element	Analyte	LOD
Immunosensor array [56]	Au electrode modified with nanogold particles	HAV monoclonal antibody	HAV antigen	0.1 pg/mL
Indirect competitive Immunosensor [29]	Carbon nano-powder paste electrode	HAV capture antibodies	HAV antigen	26 × 10^−5^ IU/mL
DNA biosensor [28]	Au electrode	ssDNA probe	HAV ssDNA and cDNA	0.65 pM for ssDNA; 6.94 fg/µL for viral cDNA
Cell biosensor (This study)	Au-modified screen-printed electrode	FRhK-4 cells	Infectious HAV	5 TCID_50_/mL

## 4. Conclusions

As summarized in Table 1, previous studies on biosensors have predominantly detected viral proteins, nucleic acids, or antigens; the cell-based approach enables direct measurement of the interaction between HAV and host cells, closely mimicking natural biological processes of viral infection. Taken together, these results highlight the potential of the developed cell-based biosensor for rapid and sensitive detection of infectious HAV. The use of FRhK-4 cells as biological receptors, combined with the enhanced electrode surface provided by gold nanoparticles, likely contributed to the high sensitivity and specificity observed. The biosensor is stable for at least 3 days under an abusive temperature (37 °C). This cell-based biosensor approach for HAV addresses the limitations of traditional cell culture infectivity assays, such as TCID_50_ assay, by providing a faster (6 h versus 10–14 days) and reliable alternative for the detection of infectious HAV. Also, the biosensor can effectively distinguish between infectious and non-infectious HAV. The ability to quickly detect infectious HAV at low LOD (~0.7 log equivalent to ~5 particles TCID_50_/mL) can significantly impact the management of an HAV outbreak, enabling early intervention and reducing the spread of the virus. Future applications of the developed prototype cell-based biosensor will include testing in various food, water, and environmental matrices to optimize this biosensor as a tool for food safety monitoring and public health protection. Integrating cell-based biosensors into food safety testing presents challenges, including the recovery and concentration of viruses from complex food matrices, which necessitate careful upstream method optimization to preserve viral infectivity and ensure accurate detection. Addressing these challenges is essential for the food safety application of these biosensors.

## Data Availability

Data are available upon request from authors.
